# Serum proteomics identify CSF1R as a novel biomarker for postoperative recurrence in chronic rhinosinusitis with nasal polyps

**DOI:** 10.1016/j.waojou.2024.100878

**Published:** 2024-03-02

**Authors:** Yan Niu, Shouming Cao, Maoxiang Luo, Jinmei Ning, Nanan Wen, Haiying Wu

**Affiliations:** a Department of Otolaryngology-Head and Neck Surgery, The Second Affiliated Hospital of Kunming Medical University, Kunming, People's Republic of China; b Department of Otolaryngology-Head and Neck Surgery, Yanjin County People's Hospital, Yanjing, People's Republic of China; c Department of Otolaryngology-Head and Neck Surgery, The First People's Hospital of Qujing City, Qujing, People's Republic of China

**Keywords:** Serum, Proteomics, Nasal polyps, Macrophages

## Abstract

**Background:**

Chronic rhinosinusitis with nasal polyps (CRSwNP) presents a high rate of postoperative recurrence, but its recurrent mechanisms are not fully clarified. In this study, we aim to explore biomarkers associated with the recurrence of CRSwNP and shed light on the underlying recurrent mechanisms using serum proteomics.

**Methods:**

A prospective cohort of CRSwNP patients was conducted, and serum samples were subjected to proteomic profiling. Participants were followed up for 2 years and divided into non-Recurrence and Recurrence groups and differentially expressed proteins (DEPs) were compared. The top 3 DEPs were validated in the serum and tissue samples in a validation cohort, and their predictive values for recurrence and their associations with macrophages were evaluated. *In vitro*, circulating macrophages were utilized to explore the influence of candidate proteins on macrophage polarization in underlying recurrent mechanisms of CRSwNP.

**Results:**

Sixteen CRSwNP patients completed the follow-up schedule, including 10 patients in the non-Recurrence group and 6 patients in the Recurrence group. Serum proteomics revealed a distinctive protein expression profile between the 2 groups. A validation cohort comprising 51 non-recurrent and 24 recurrent CRSwNP patients was recruited. Enzyme-linked immunosorbent assay (ELISA) results revealed that circulating levels of CSF1R and CDC42 were significantly higher, and DHRS9 levels were lower in the Recurrence group in comparison with the non-Recurrence group. In addition, tissue CSF1R and CDC42 were identified to be enhanced in the Recurrence group compared to the non-Recurrence group. Receiver-operated characteristic (ROC) curves and Kaplan-Meier survival analysis suggest that both serum and tissue CSF1R were associated with the risk of postoperative recurrence. Tissue immunofluorescence (IF) revealed that CSF1R was enhanced in the tissues of patients with recurrence, especially in the mesenchymal region. Multiplex IF highlighted that CSF1R was significantly co-expressed with M2 macrophage markers. In vitro experiments confirmed that CSF1R overexpression promoted macrophage M2 polarization and cytokine production.

**Conclusion:**

Serum proteomic signatures may affect postoperative recurrence in CRSwNP patients. CSF1R is a potential biomarker for predicting CRSwNP recurrence. Mechanistically, the recurrence of CRSwNP appears to involve the CSF1R-driven M2 polarization process.

## Introduction

Chronic rhinosinusitis (CRS) is a prevalent chronic inflammatory mucosal condition within the field of otolaryngology.^1^ Recent epidemiological research indicates that the worldwide prevalence of CRS exceeds 10% and continues to rise annually.^2^^,^^3^ Based on the presence or absence of polyps at nasal endoscopy, CRS has 2 phenotypes, CRS without nasal polyp (CRSsNP) and CRS with nasal polyp (CRSwNP).^4^ Accordingly, CRSsNP is mainly characterized by Th1-type inflammation, while CRSwNP is primarily characterized by Th2-type inflammation.^5^^,^^6^ Previous research indicated that variations in inflammatory patterns influence the clinical manifestations, disease severity, and prognosis, and CRSwNP exhibits more complex tissue heterogeneity and a higher postoperative recurrence rate compared to CRSsNP.^7^^,^^8^ Recent research findings indicated that individuals with CRSwNP experienced a notably high postoperative recurrence rate, even reaching up to 55.3%, often necessitating subsequent revision surgery.^9^, ^10^, ^11^ The postoperative recurrence of CRSwNP poses significant challenges to clinical management and greatly impacts the patients' quality of life, exacerbating the personal and societal economic burden Therefore, exploration of underlying mechanisms of recurrent CRSwNP and identification of biomarkers for predicting postoperative recurrence holds significance in advancing disease prognosis, refining clinical decisions, and shedding light on the mechanisms underlying disease recurrence.

Serum proteomics, as an emerging research technique in the field of biomedical research, involves the comprehensive analysis of proteins to identify biomarkers, understand disease mechanisms, and enhance disease diagnosis and prognosis.^12^^,^^13^ In a study by Cheng et al^14^ serum proteomics was employed utilizing high-resolution mass spectrometry analysis to construct a serum protein profile. They identified 18 proteins that were significantly overexpressed. Notably, FCN-2 protein levels were found to be elevated in rheumatoid arthritis and exhibited a correlation with disease activity. Mun et al^15^ suggested that the protein S100A9 identified by serum proteomics was involved in the destructive and pro-inflammatory response of matrix metalloproteinases to joints and could potentially be used as a diagnostic biomarker reflecting the inflammatory mechanisms of rheumatoid arthritis. Moreover, serum proteomic signatures have proven to be pivotal in the underlying mechanisms of airway inflammatory diseases.^16^ A recent study discovered through serum proteomics that the expression of LDHA and CCT6A was significantly elevated in the serum of individuals with pulmonary fibrosis. These proteins were identified as potential biomarkers capable of accurately distinguishing individuals with pulmonary fibrosis from healthy subjects.^17^ However, there has been limited research on serum proteomics in patients with CRSwNP, particularly regarding its relationship with disease recurrence, which remains poorly understood.

To address this knowledge gap, we performed a prospective study and conducted serum proteomic analysis to explore potential serum proteins associated with CRSwNP recurrence. Our data showed that CRSwNP patients who suffered recurrence exhibited distinctive serum proteomic profiles compared to those who did not suffer recurrence. Specifically, we found that both baseline serum and tissue CSF1R levels were associated with the risk of postoperative recurrence. Mechanistically, we demonstrated that CSF1R facilitated the macrophage M2 polarization process contributing to the recurrence of CRSwNP.

## Methods and materials

### Patients and settings

This prospective study was approved by the ethical committee of our hospital (No. 2023231). All participants signed informed consent. We recruited 2 independent cohorts between July 2019 and September 2019, including a discovery cohort and a validation cohort. The discovery cohort comprised initially 18 patients with CRSwNP who underwent functional endoscopic sinus surgery (FESS) in our department. Additionally, 80 CRSwNP patients treated with FESS were recruited for the validation cohort. For all enrolled patients, the diagnosis of CRSwNP was made according to the EPOS 2012.^18^ Exclusion criteria included:^1^ age<18 years;^2^ concurrent comorbidities such as fungal sinusitis, posterior nasal polyps, sinus cysts, and acute respiratory infections; and^3^ history of treatment with antibiotics, steroids, antihistamines, and leukotriene receptor antagonists within 4 weeks. We collected baseline clinical data, encompassing gender, age, body mass index (BMI), and comorbidities. Preoperative CT scans and nasal endoscopy results were assessed using the Lund-Mackay and Lund-Kennedy scoring systems, respectively.^11^

### Follow-up and postoperative recurrence assessment

In both the discovery and validation cohorts, patients diagnosed with CRSwNP underwent FESS performed by skilled surgeons following standardized protocols. A uniform postoperative care plan was administered to all patients, including nasal saline irrigation, oral antibiotics, and topical corticosteroids, in line with established guidelines.^19^ The patients were actively monitored for over 2 years, during which regular nasal endoscopic assessments were conducted. The recurrence of CRSwNP was defined by the reappearance of clinical symptoms and persistent endoscopic evidence lasting at least 2 months, despite undergoing a rescue regimen of antibiotics and oral steroids, as previously described.^9^ Based on the follow-up results, patients were categorized into either the Recurrence or non-Recurrence groups.

### Clinical samples and serum proteomic analysis

Serum samples were harvested from all CRSwNP patients. The serum proteomic profiles were analyzed utilizing the liquid chromatography-mass spectrometry (LC-MS) label-free system as previously described.^20^ SDT buffer was added to the serum samples to collect peptides. For each sample, 200 ng of protein was loaded directly onto a 15 cm long Bruker nanoElute FIFTEEN C18 analytical column (Bruker) and analyzed in a gradient at 400 nL/min for 30 min. The column was heated to 50°C using an oven. Proteins were identified and quantified using MaxQuant software with default settings as previously described.^20^^,^^21^ We conducted searches against a database containing human protein entries (Uniprot/Swissprot). Tolerances of 20 ppm and 0.05 Da were applied for precursors and fragments, respectively. Proteins with a minimum of 2 identified peptides at a false discovery rate (FDR) < 1% were selected for further analysis. The data-dependent acquisition (DDA) was performed in positive mode using the Spectronaut System (Biognosys, Switzerland) according to a predefined protocol.^22^ A dynamic exclusion time window of 45 s was set, and ions with a single charge or a charge greater than 6 were excluded from the DDA process as previously described.^20^

### Bioinformatic analysis

Differentially expressed proteins (DEPs) between the Recurrence and non-Recurrence groups were identified with P value < 0.05 and fold change >1.5, and they were visually displayed using R packages through heat maps and volcano maps. To identify potential proteins contributing to the proteomic differences between the 2 groups, principal component analysis (PCA) was constructed. To explore the biological processes associated with DEPs, Gene Ontology (GO) pathway analysis and Kyoto Encyclopedia of Genes and Genomes (KEGG) were performed.

### Enzyme-linked immunosorbent assay (ELISA)

The top 3 DEPs detected by serum proteomics were chosen for validation in the validation cohort with enzyme-linked immunosorbent assay (ELISA) according to the manufacturer's instructions. The concentrations of cytokines in the cell supernatants were also detected with ELISA. Colony-stimulating factor 1 receptor (CSF1R), C–C motif chemokine 5 (CCL5), interleukin (IL)-10, tumor necrosis factor (TNF)-α, IL12, and transforming growth factor (TGF)-β ELISA kits were provided by Cusabio (Wuhan, China). Cell division control protein 42 (CDC42) ELISA kits, ubiquitin-conjugating enzyme E2 variant 1 (UBE2V1) ELISA kits, pregnancy zone protein (PZP) ELISA kits, dehydrogenase/reductase (DHRS9) ELISA kits were provided by ARP Inc™ (Waltham, USA). Absorbance was measured at 450 nm using a microplate reader. The operators performing the assay were kept blinded to the specific patient data.

### Quantitative reverse transcription polymerase chain reaction (qRT-PCR)

Tissue specimens were collected during the surgery. Total RNA was extracted from the tissues using a TRIzol reagent (Invitrogen, USA). cDNA was synthesized from 1 μg of total RNA using a reverse transcription kit (Qiagen, Germany). Quantitative PCR was performed using the ABI PRISM 7300 Detection System (Applied Biosystems, USA) with SYBR Premix EX Taq (Suzhou, China). Primer sequences are listed in Table S1. Glyceraldehyde-3-phosphate dehydrogenase (GAPDH) was selected as a housekeeping gene to normalize gene expression. We analyzed the relative mRNA levels of the target genes using the comparative threshold cycling (2-ΔΔCt) method.

### Western blotting (WB)

Proteins from the tissues were extracted by employing RIPA lysis buffer, and their concentrations were assessed through the utilization of a BCA protein assay kit (Beyotime, China). Proteins were treated with loading buffer and electrophoresed on SDS polyacrylamide gels and then transferred to polyvinylidene difluoride membranes. After incubation with skim milk for 1 h, the membranes were incubated overnight at 4° refrigerator with the corresponding primary antibody against CSF1R, CDC42, DHRS9, CD86, NOS2, CD163, and CD206 (Abcam, USA). The membranes are then incubated with the secondary antibody (Abcam, USA). Protein bands were detected by ECL ultrasensitive luminescent phenol (Beyotime, China), and their intensities were measured by Image J software. The target proteins were normalized to β-actin (Abcam, USA) to determine the expression levels of the target proteins.

### Immunofluorescence (IF) analysis

Immunofluorescence (IF) was performed as described previously.^23^ Briefly, paraffin-embedded sections of collected nasal polyp tissues were baked at 65°C for 3 h, then deparaffinized and rehydrated with xylene and ethanol, respectively. After soaking in boiling citrate buffer (0.01 M, pH 6.0) for 10 min and blocking with 10% goat serum for 30 min, the sections were incubated with anti-CSF1R, anti-CDC42, and anti-DHRS9 (Abcam, USA) antibodies at 4°C overnight. The primary antibody was removed and washed with PBS 3 times, and then 2 rounds of staining were carried out using fluorescence-conjugated secondary antibodies (Abcam, USA). Cell nuclei were stained with DAPI (Beyotime, China). The triple fluorescence staining was carried out using the Dual-label Multiplex Immunoassay Kit (Aifang, China) following the provided protocol. Initially, tissue sections were incubated with primary antibodies against CSF1R overnight at 4C, followed by incubation with horseradish peroxidase (HRP) labeled secondary antibody (Abcam, USA). After washing, antigen retrieval was performed, and the sections were incubated with CD86 (CD206) primary antibody (Abcam, USA) overnight at 4°C. Subsequently, the sections were incubated with Cy3-labeled fluorescent secondary antibody (Abcam, USA) for 1 h. DAPI solution was added to each slice to visualize the nuclei. All slides were then covered with glass and observed under a fluorescence microscope. The analysis involved assessing relative fluorescence expression and the number of positive cells under high-power fields (HPF, × 400).

### Culture and transfection of peripheral blood macrophages

Isolation of human peripheral blood macrophages was performed as previously described.^24^ Peripheral blood mononuclear cells (PBMCs) were treated with anti-CD14 magnetic beads (Miltenyi, China) to sort out monocytes. After isolation and sorting, monocytes were cultured in RPMI-1640 medium (Gibco, China) supplemented with 10% fetal bovine serum (Gibco, USA). To induce macrophage differentiation, M-CSF (Prospect, UK) at a concentration of 100 ng/ml was added and then cultured at 37°C and 5% CO2 for 5 days as previously outlined.^25^ The resulting PBMC-derived macrophages were collected and further cultured. For transient transfection, macrophages were transfected with CSF1R over-expression (OE) plasmid (Ruibo, China) using the reagent Lipofectamine 3000 (Invitrogen, USA). The treated cells and cell supernatants were collected for subsequent experiments.

### Statistical analysis

Numerical data were presented as mean ± standard deviation for variables with a normal distribution, and Student's t-test was utilized for comparison. For variables that were not normally distributed, median and interquartile ranges (IQRs) were reported, and the Mann-Whitney *U* test was employed. Categorical data were expressed as frequencies and percentages, and differences were compared using the chi-square test. In the case of experimental data, mean ± standard error of the mean (SEM) was provided, and the Mann-Whitney *U* test was applied. Receiver operating characteristic curve (ROC) analyses were conducted to assess the predictive potential of proteins in CRSwNP recurrence. Patients were categorized into high and low-level groups based on the median values of serum or tissue protein levels. Kaplan-Meier survival analysis was performed to evaluate the association between protein levels and the risk of CRSwNP recurrence. All statistical analyses were performed on SPSS statistical software. A significance level of 0.05 was considered for all tests, and p-values below this threshold were deemed statistically significant.

## Results

### Clinical characteristics of all subjects

In the discovery cohort, a total of 16 CRSwNP patients completed the whole follow-up schedule and were included in the serum proteomic analysis, including 10 patients in the non-Recurrence group, and 6 patients in the Recurrence group. There were no notable differences in gender, age, BMI, allergic rhinitis, asthma, Lund-MacKay score, and Lund-Kennedy score between the 2 groups (all P > 0.05), except for variations in follow-up time (Table S2).

### Distinct proteomic profiles associate with CRSwNP recurrence

**Fig. 1:**
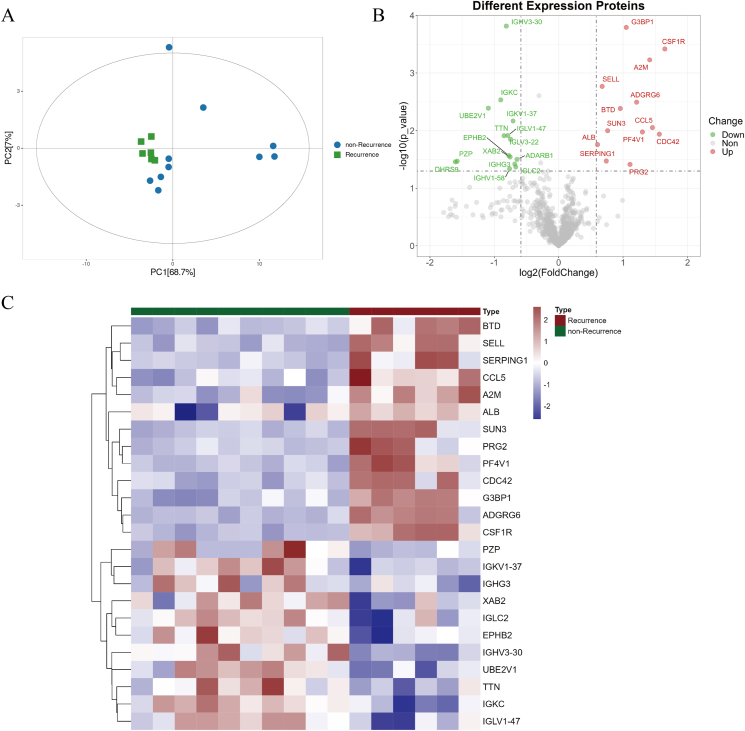
Serum proteomic identified a distinctive protein profile in CRSwNP patients between the Recurrence and non-Recurrence groups. (A) PCA model of the proteomics data between the 2 groups; (B) volcano; (C) heatmap. CRSwNP, chronic rhinosinusitis with nasal polyp. PCA, principal component analysis

**Fig. S1:**
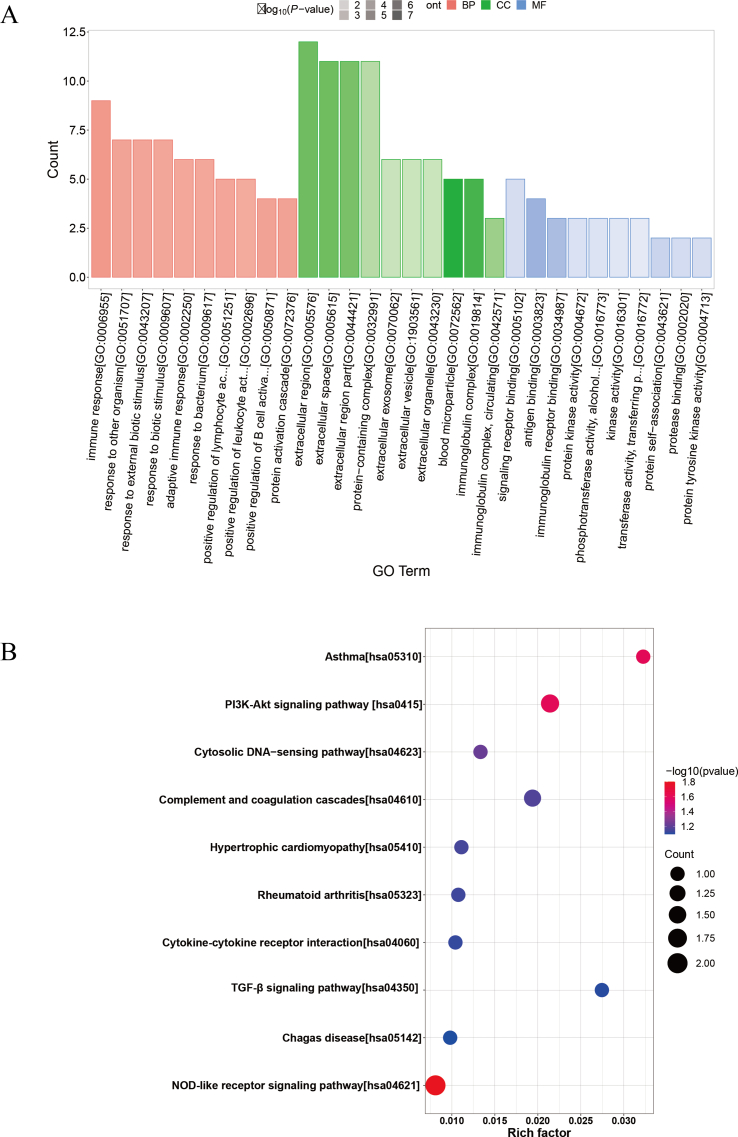
GO and KEGG pathway enrichment analysis of DEPs between the Recurrence and non-Recurrence groups. (A) GO; (B)KEGG. CRSwNP, chronic rhinosinusitis with nasal polyps; GO, Gene Ontology; KEGG, Kyoto Encyclopedia of Genes and Genomes

Based on proteomic analysis, the results of protein identification and quantification are illustrated in Fig. 1. Principal component analysis revealed clear and independent clustering of samples between the non-Recurrence and Recurrence CRSwNP groups (Fig. 1A). Further heatmaps and volcano plots indicated significant differences in protein expression patterns between these 2 groups (Fig. 1B–C). The GO annotations and pathway enrichment analysis of the DEPs are depicted in Figure S1A. Additionally, the KEGG pathway and enrichment analyses revealed that the differentially expressed proteins primarily played a role in the development of asthma and the PI3K-Akt signaling pathway. (Figure S1B).

### Elevated CSF1R facilitates the risk of postoperative recurrence in CRSwNP

**Fig. 2:**
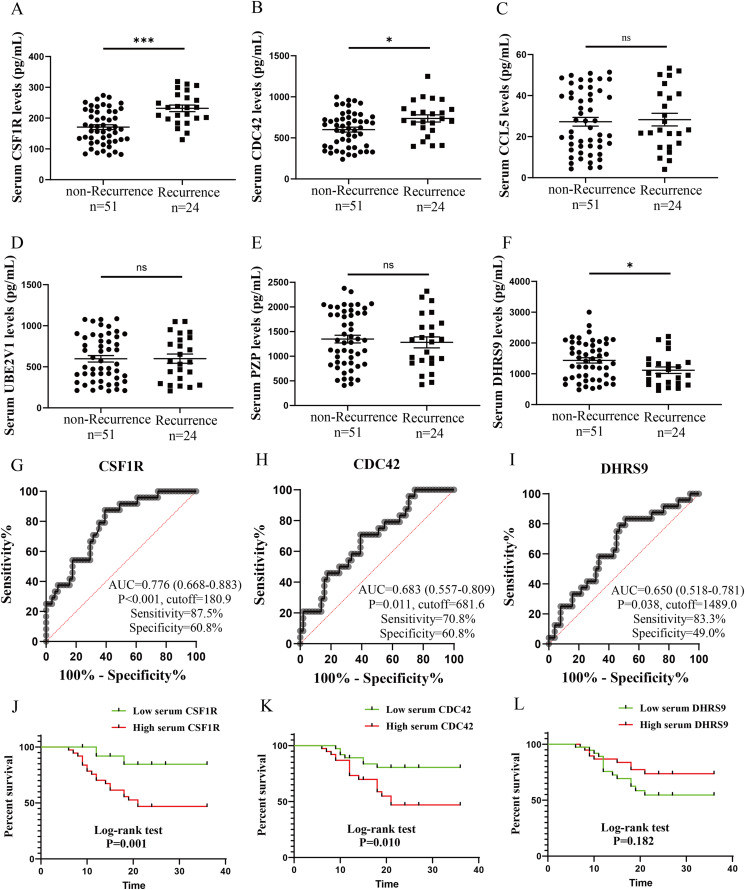
Validating serum concentrations of the potential DEPs and exploring the predictive values for CRSwNP recurrence in the validation cohort. (A) CSF1R; (B) CDC42; (C) CCL5; (D) UBE2V1; (E) PZP; (F) DHRS9. (G–I) ROC curves; (J–L) Kaplan-Meier survival analysis. ROC, receiver operator characteristic; CRSwNP, chronic rhinosinusitis with nasal polyps. ∗P < 0.05; ∗∗∗P < 0.001; ns, no significance

The top 3 DEPs listed in Table S3 were chosen to be validated in a validation cohort. The validation cohort finally comprises 51 patients with non-Recurrence and 24 patients with Recurrence after 2 years of follow-up (Table 1). The ELISA results in Fig. 2A–F demonstrated that serum levels of CSF1R and CDC42 were significantly elevated, while DHRS9 levels were decreased in the Recurrence group compared to the non-Recurrence group (P < 0.05). The ROC curves and Kaplan-Meier suggested that serum CSF1R exhibits more excellent predictive values for postoperative recurrence compared to the other 2 proteins (Fig. 2G-L).

**Table 1:** Characteristics of CRSwNP patients in the validation cohort.

	non-Recurrence	Recurrence	P
Number, (n)	51	24	
Male, n (%)	34 (66.7)	14 (58.3)	0.607
Age, years	43.0 (31.0, 48.0)	46.0 (34.5, 50.0)	0.277
BMI, kg/m^2^	23.0 (21.0, 25.4)	23.5 (21.3, 26.7)	0.302
Allergic rhinitis, n (%)	14 (27.5)	8 (33.3)	0.599
Asthma, n (%)	5 (9.8)	6 (25.0)	0.158
Lund-MacKay score	13.0 (10.0, 15.0)	14.0 (12.0, 15.0)	0.687
Lund-Kennedy score	7.0 (5.0, 8.0)	7.0 (6.0, 8.0)	0.892
Follow-up time, months	24.0 (18.0, 24.0)	12.0 (9.0, 18.0)	<0.001

CRSwNP, chronic rhinosinusitis with nasal polyps; BMI, body mass index; VAS, visual analogue score

**Fig. 3:**
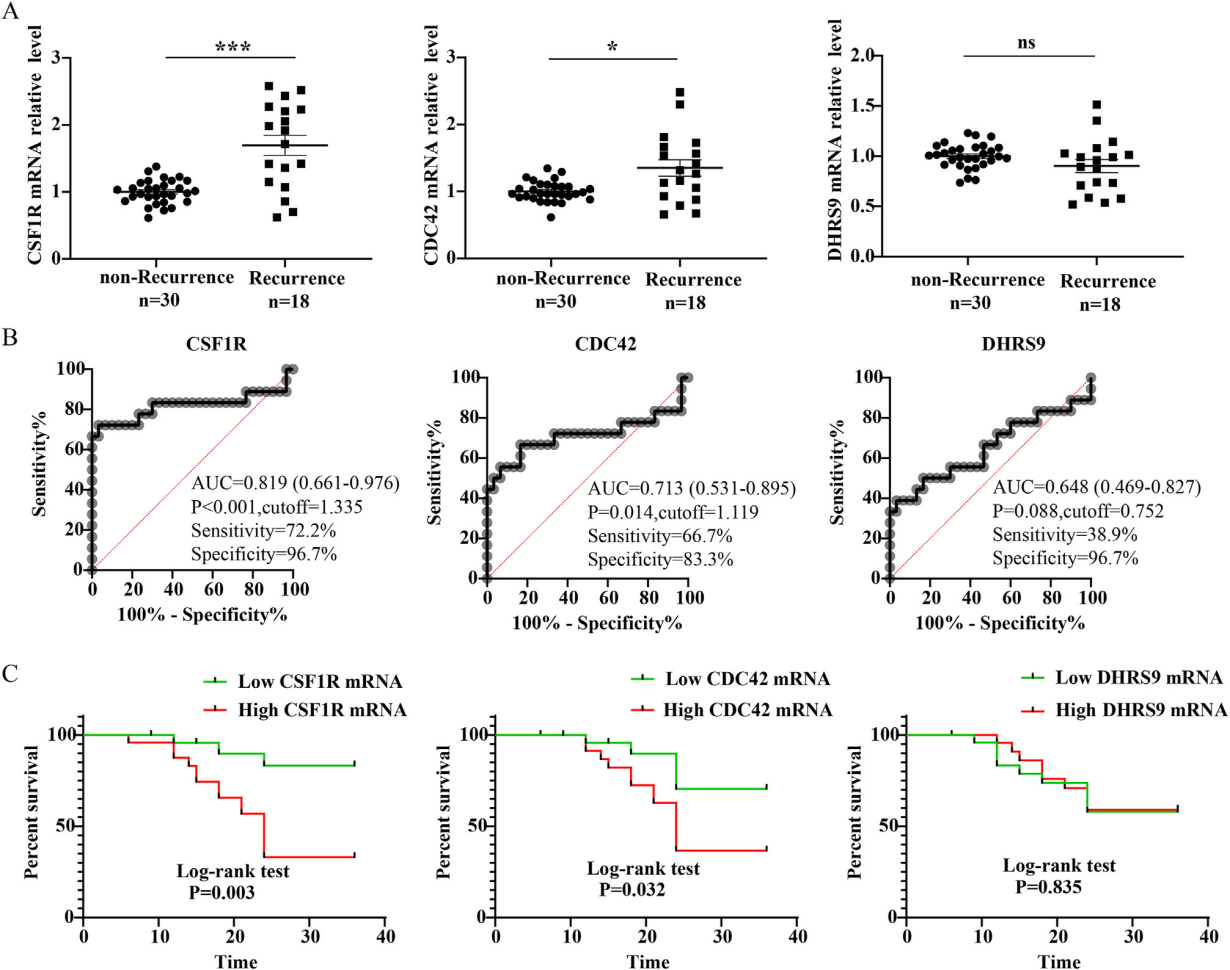
Tissue mRNA expressions of 3 candidate proteins in CRSwNP and their associations with the risk of postoperative recurrence. (A) tissue mRNA expressions of 3 candidate proteins between the 2 groups; (B) ROC curves; (C) Kaplan-Meier survival analysis. ROC, receiver operator characteristic. CRSwNP, chronic rhinosinusitis with nasal polyps. ∗P < 0.05; ∗∗∗P < 0.001; ns, no significance

**Fig. 4:**
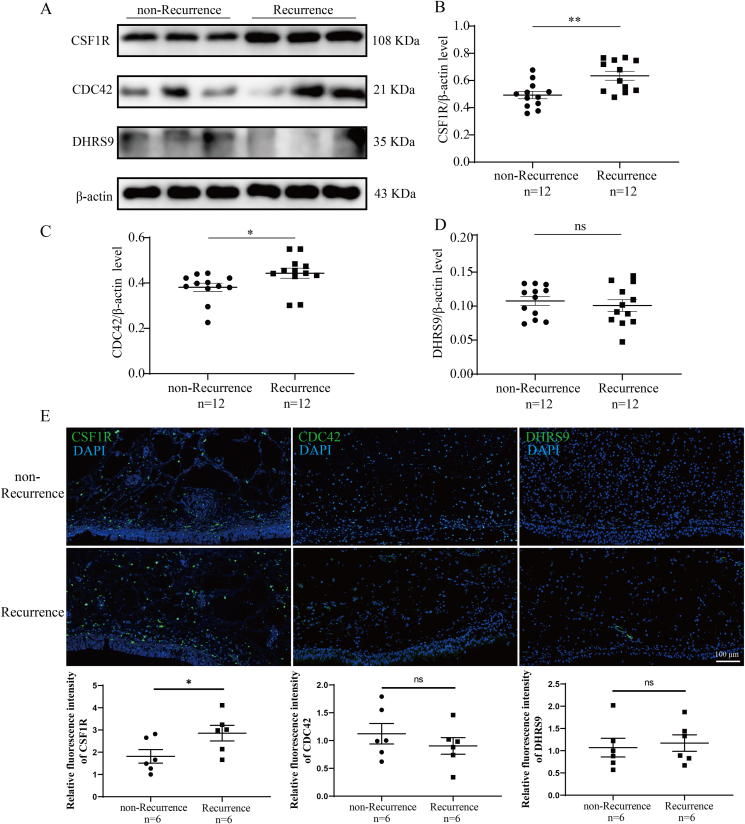
Tissue protein expression of 3 candidate proteins between the Recurrence and non-Recurrence groups. (A) representative images of WB; (B–D) relative protein levels of the 3 candidate proteins between the 2 groups; (E) immunofluorescence staining in the tissues between the 2 groups. WB, western blotting. ∗P < 0.05; ∗∗P < 0.01; ns, no significance

To delve deeper into the connection between candidate proteins and the postoperative recurrence of CRSwNP, we evaluated the expressions of CSF1R, CDC42, and DHRS9 in nasal polyp tissues. As depicted in Fig. 3, qRT-PCR results demonstrated a significant increase in CSF1R and CDC42 expression in the Recurrence group compared to the non-Recurrence group (P < 0.05). ROC curves and Kaplan-Meier curves indicated a correlation between tissue CSF1R and CDC42 mRNA levels and the risk of postoperative recurrence (P < 0.05). Furthermore, the WB and IF results revealed an augmented tissue CSF1R protein level in the Recurrence group compared to the non-Recurrence group (P < 0.05), with fluorescence intensity primarily concentrated in the mesenchymal regions (Fig. 4). These data implied that elevated CSF1R expression might play a crucial role in the underlying mechanisms of recurrent CRSwNP.

### CSF1R-driven macrophage M2 polarization contributes to CRSwNP recurrence

**Fig. 5:**
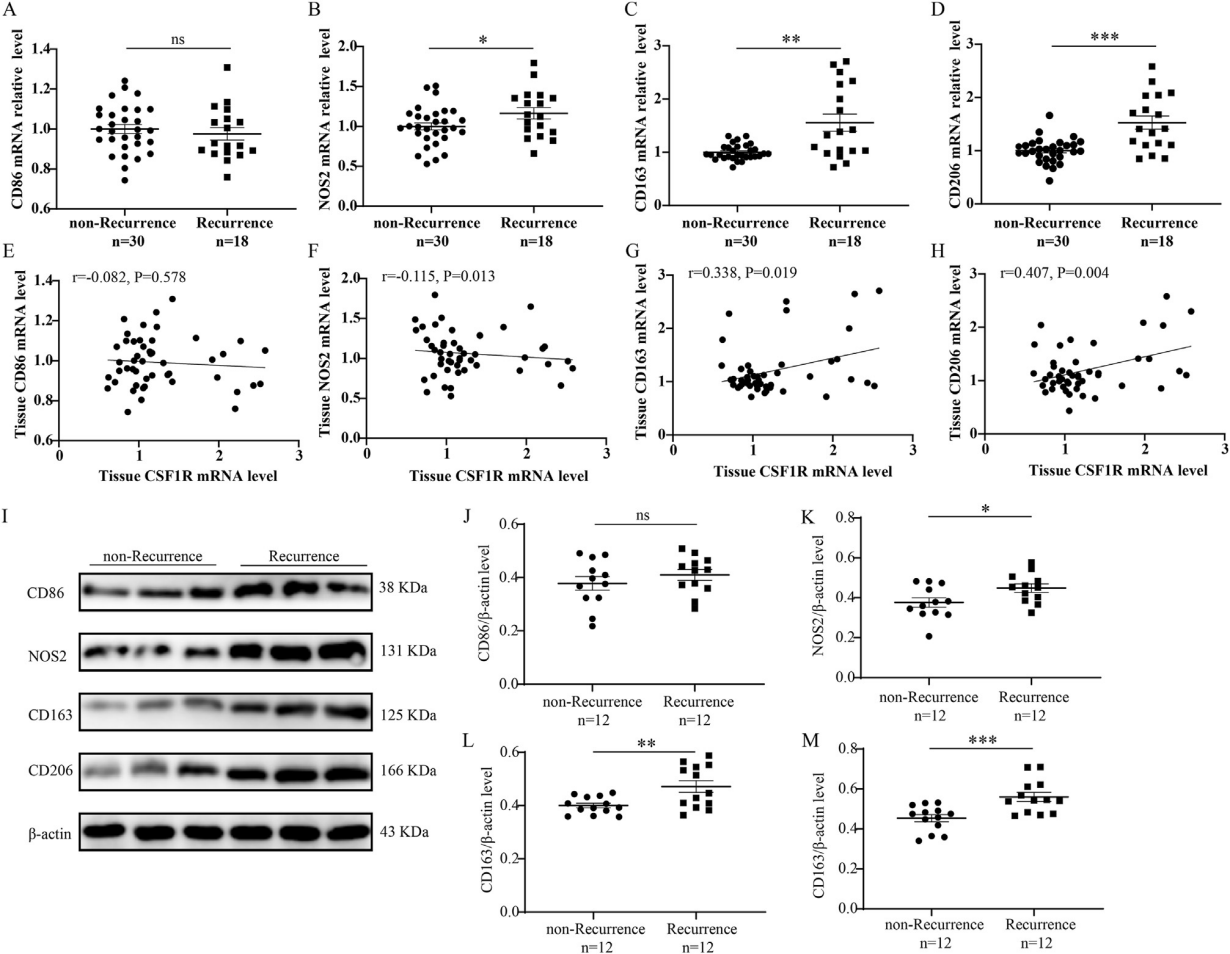
Tissue expression of macrophage polarization markers in recurrent group and non-recurrent groups and correlation with CSF1R. (A–D) Tissue mRNA levels of macrophage polarization markers between the 2 groups. (E–H) The correlation between the tissue mRNA levels of macrophage polarization markers and CSF1R mRNA levels. (I–M) Tissue protein levels of macrophage polarization markers between the 2 groups. ∗P < 0.05; ∗∗P < 0.01; ∗∗∗P < 0.001; ns, no significance

**Fig. 6:**
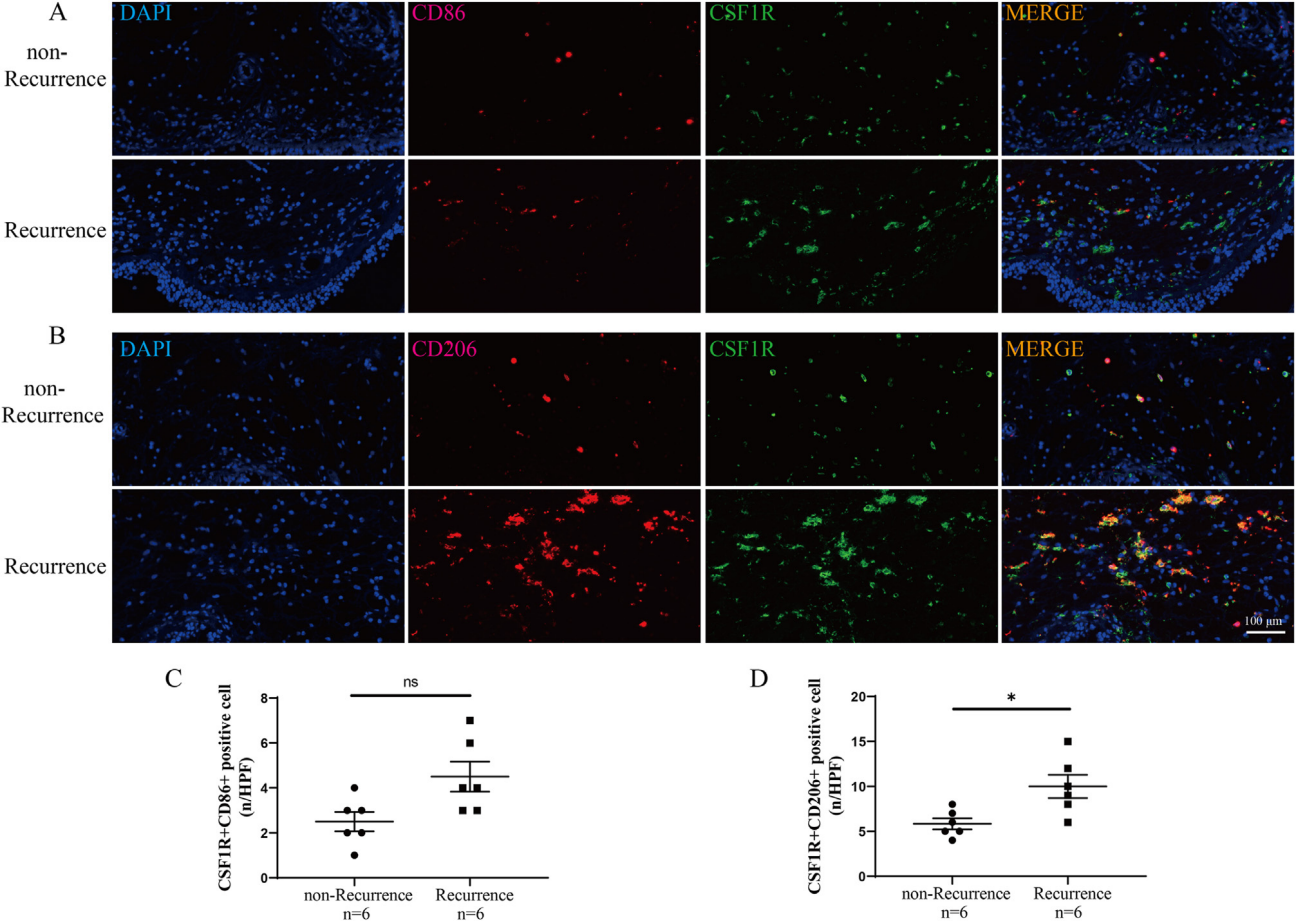
Multiplex immunofluorescence staining evaluates the co-expressions of CSF1R and macrophage markers between the 2 groups. (A) representative images of co-expression of CSF1R and CD86; (B) representative images of co-expression of CSF1R and CD206; (C) comparison of positive cell number of CSF1R + CD86^+^; (D) comparison of positive cell number of CSF1R + CD206+. ∗P < 0.05; ns, no significance

**Fig. 7:**
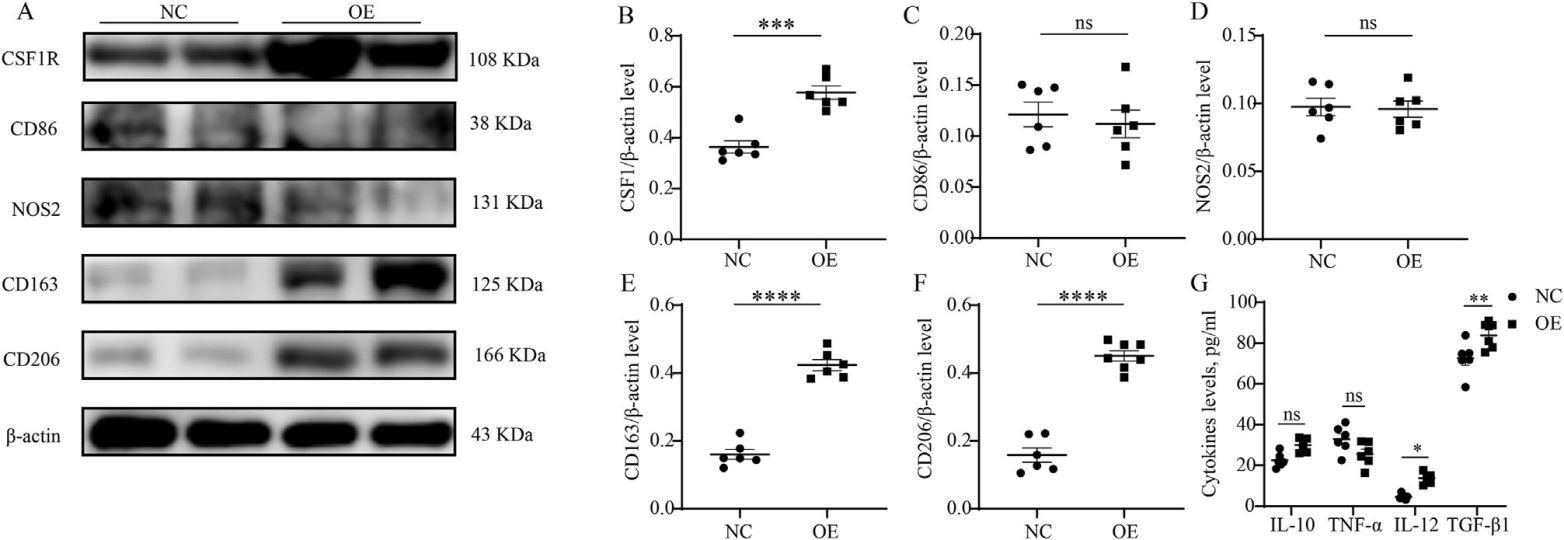
The influence of CSF1R overexpression on macrophage polarization. (A–F) WB shows the protein levels of macrophage markers between the OE and the NC groups. (G) the comparisons of cytokine levels in the cell supernatants between the OE and the NC groups. ∗P < 0.05, ∗∗P < 0.01, ∗∗∗P < 0.001, ∗∗∗∗P < 0.0001. NC, normal control; OE, overexpression

Previous studies demonstrated that CSF1R could affect macrophage function, and macrophage polarization was critical in the pathogenesis of CRSwNP.^26^^,^^27^ Therefore, we detected the expressions of macrophage polarization markers in the tissue samples of CRSwNP. We found that tissue NOS2, CD163, and CD206 were increased in the Recurrence group compared to the non-Recurrence group (P < 0.05), and tissue CSF1R mRNA levels positively correlated with the expressions of CD163 and CD206 (Fig. 5A–H, P < 0.05). WB results supported the elevations of tissue NOS2, CD163, and CD206 in the Recurrence group compared to the non-Recurrence group (P < 0.05). Multiplex immunofluorescence results in Fig. 6 revealed that CSF1R and CD206 were co-expressed in tissues of CRSwNP, the numbers of CSF1R + CD206+ -positive cells were elevated in the Recurrence group compared to the non-Recurrence group (P < 0.05). These findings suggested that M2 polarization was prevalent in the histopathology of CRSwNP and CSF1R-mediated M2 polarization might be involved in the recurrent mechanism. To further explore the influence of CSF1R on M2 polarization, we employed CSF1R OE-plasmids to treat macrophages. The WB results demonstrated a significant increase in the expressions of CD163 and CD206 with up-regulation of CSF1R, while there was minimal effect on NOS2 and CD86 levels (Fig. 7A–F). Importantly, the heightened expression of CSF1R in macrophages led to increased secretion of IL-12 and TGF-β1 in the cell supernatants (Fig. 7G).

## Discussion

Currently, the precise mechanisms triggering postoperative recurrence in CRSwNP remain elusive, presenting a significant challenge in clinical management. Although recent studies have advanced our understanding of the mechanisms involved in recurrent CRSwNP and shed light on postoperative recurrence mechanisms. These studies have delved into various aspects, including circulating and tissue cytokines,^28^ metabolites,^9^^,^^29^ and nasal microbiome.^30^ However, despite these advancements, the complexities underlying postoperative recurrence mechanisms remain not completely elucidated. Hence, there is a critical need to develop a practical and reliable approach for identifying concrete biomarkers that can accurately predict the risk of recurrence.

In this prospective study, we conducted serum proteomics in the discovery cohort and successfully identified significant associations between baseline serum proteomic profiles and the risk of postoperative recurrence. The top DEPs were validated in an independent validation cohort, affirming that serum CSF1R and CDC42 hold predictive values for postoperative recurrence in CRSwNP. Additionally, heightened baseline tissue levels of CD40 and CSF1 were linked to an increased risk of postoperative recurrence. Notably, tissue expression of CSF1R was positively correlated with CD163 and CD206 levels, indicating a potential role in promoting macrophage M2 polarization. Our mechanistic insights suggest that CSF1R overexpression can enhance macrophage M2 polarization and cytokine production, potentially contributing to recurrence in CRSwNP. Additionally, CSF-1R holds the potential to serve as an objective clinical biomarker for predicting early postoperative recurrence in CRSwNP. It is also anticipated to be a potential target for the treatment of recurrent CRSwNP.

CSF1R, a receptor protein expressed in diverse cell types such as epithelial cells and macrophages, plays a crucial role in regulating their development and differentiation. This regulatory function contributes to the pathological processes observed in various diseases.^31^^,^^32^ Recent studies found that CSF1R was involved in the inflammatory process of asthma by regulating the number and function of macrophages, and activated the CSF1R signaling pathway and might lead to macrophage accumulation and excessive inflammatory response, exacerbating asthma symptoms.^33^^,^^34^ In another study, it was observed that CSF1R played a role in sustaining airway inflammation by promoting the recruitment and activation of macrophages and other inflammatory cells. This activity-induced airway remodeling, results in a narrowed airway and restricted airflow.^35^ However, the expression levels and the specific mechanisms involving CSF1R in patients with CRSwNP have yet to be fully elucidated. In this study, we observed elevated levels of CSF1R in the serum and tissues of patients with CRSwNP, and CSF1R levels were associated with the risk of postoperative recurrence in CRSwNP. Interestingly, we noted that CSF1R was selectively expressed on tissue CD206+ macrophages, especially in recurrent CRSwNP, and up-regulation of CSF1R aggravated the M2 polarization and cytokine productions.

Macrophages are important members of the innate immune cells, which are highly plastic and heterogeneous and have an important role in airway inflammatory responses.^36^, ^37^, ^38^ Previous research indicated that macrophage polarization played a crucial role in tissue repair and remodeling. This process involved the secretion of diverse cytokines and inflammatory mediators such as IL-10 and TGF-β1, ultimately contributing to pathological tissue remodeling.^39^^,^^40^ Recently, growing evidence suggested that macrophage polarization patterns were involved in the pathogenesis of CRSwNP and associated with its tissue heterogeneity.^26^^,^^41^ However, the precise role of macrophage polarization in recurrent CRSwNP and the regulatory mechanisms driving it remains incompletely understood. In this study, we demonstrated a significant increase in expression of M2 markers (CD206 and CD163) in recurrent CRSwNP, indicating a dominant M2 polarization in recurrent pathology.

Previous research has demonstrated that CSF1R can promote macrophage polarization and drive tissue remodeling, potentially exacerbating disease severity and influencing prognosis.^42^ It has been reported that recurrent CRSwNP was usually characterized by tissue remodeling, including epithelial-mesenchymal transition, fibrosis, and tissue eosinophilia.^8^^,^^43^^,^^44^ Fascinatingly, macrophage M2 polarization has a dual impact: it exacerbates tissue remodeling by releasing inflammatory mediators that aid in tissue repair while disrupting the normal epithelial mucosal barrier.^45^ Simultaneously, it amplifies eosinophilic inflammation by recruiting and facilitating the migration of eosinophils into tissues.^46^^,^^47^ To explore the role of CSF1R in driving M2 polarization and its association with CRSwNP recurrence, we conducted an overexpression of CSF1R in macrophages. We observed that up-regulation of CSF1R enhanced macrophage M2 polarization and secretion of IL-12 and TGF-β1. Therefore, we propose the hypothesis that external stimuli such as bacteria, viruses, and antigens in the nasal mucosa promote the release of cell factors, including CSF1. CSF1 binds to CSF1R on the surface of macrophages, promoting macrophage M2 polarization and the secretion of cytokines like IL-12 and TGFβ1. This, in turn, recruits eosinophil infiltration into tissues, while also acting on the nasal mucosa to promote tissue remodeling, mediating postoperative recurrence in CRSwNP. Furthermore, CSF1 can enhance the expression of CSF1R in macrophages, and higher levels of CSF1R can be released into the bloodstream in a soluble form, making it detectable as a peripheral blood biomarker.

This study has several limitations. Firstly, it was performed at a single medical center with a relatively small sample size, potentially introducing selection bias. Secondly, the relatively short follow-up duration may weaken the robustness of our findings. Thirdly, the study was limited to tissues and cells, lacking animal experiments to provide a more comprehensive understanding of CSF1R-driven macrophage M2 polarization in the recurrent mechanisms of CRSwNP.

## Conclusion

In this prospective study, we proposed an innovative method to predict postoperative recurrence in CRSwNP using serum proteomic profiling. The findings suggested that baseline circulating protein signatures exhibited a potential influence on the risk of postoperative recurrence. Our discovery-validation results suggested that CSF1R could potentially serve as objective biomarkers to predict postoperative recurrence in CRSwNP, and CSF1R-driven macrophage M2 polarization played an important role in the recurrent mechanism of CRSwNP.

## Abbreviations

CRSwNP, chronic rhinosinusitis with nasal polyps; DEP, differentially expressed proteins; ELISA, enzyme-linked immunosorbent assay; ROC, receiver-operated characteristic; IF, immunofluorescence; CRS, chronic rhinosinusitis; CRSsNP, chronic rhinosinusitis without nasal polyps; FESS, functional endoscopic sinus surgery; BMI, body mass index; LC-MS, liquid chromatography-mass spectrometry; GO, Gene Ontology; Kyoto Encyclopedia of Genes and Genomes; FDR, false discovery rate; DDA, data-dependent acquisition; CSF1R, colony-stimulating factor 1 receptor; CCL5, C–C motif chemokine 5; IL, interleukin; TNF, tumor necrosis factor; TGF, transforming growth factor; CDC42, cell division control protein 42; UBE2V1, ubiquitin-conjugating enzyme E2 variant 1; PZP, pregnancy zone protein; DHRS9, dehydrogenase/reductase; WB, western blotting; qRT-PCR, quantitative reverse transcription polymerase chain reaction; HRP, horseradish peroxidase; HPF, high-power fields; PBMC, peripheral blood mononuclear cells; OE, over-expression; IQR, interquartile ranges; SEM, standard error of the mean.

## Funding source

This research was supported by the Yunnan Provincial Department of Science and Technology-10.13039/501100003996Kunming Medical University Joint Special Project (202201AY070001-117 and 202201AY070001-122).

### Availability of data and material

All data generated or analyzed during this study are included in this published article and its Supplemental file. More related data of the current study are available from the corresponding author upon reasonable request.

## Author contribution

(I) Conception and design: Yan Niu, Shouming Cao, and Haiying Wu.

(II) Administrative support: Yan Niu, Shouming Cao, and Haiying Wu.

(III) Collection and assembly of data: Nannan Wen, Jinmei Ning, and Maoxiang Luo.

(IV) Data analysis and interpretation: Nannan Wen, Jinmei Ning, and Maoxiang Luo.

(V) Manuscript writing: All authors.

(VI) Final approval of manuscript: All authors.

## Ethics approval

This study was approved by the ethical committee of the Second Affiliated Hospital of Kunming Medical University (No. 2023231). All participants signed informed consent.

## Consent for publication

All authors have seen and approved the last version and agreed to the publication of the work.

## Declaration of competing interest

There are no patents, products in development, or marketed products to declare.
