# Supplementary material for: Serum proteomics identify CSF1R as a novel biomarker for postoperative recurrence in chronic rhinosinusitis with nasal polyps
Source: World Allergy Organ J. 2024 Mar 2;17(3):100878. doi: 10.1016/j.waojou.2024.100878 (PMC10914524; doi:10.1016/j.waojou.2024.100878)
Supplement: Multimedia component 1 [file mmc1.docx]

| Gene | Forward primer | Reverse primer |
| --- | --- | --- |
| CSF1R | TGTGTGTCCTTAGCCCCTCCCTTGC | TCAGAGCTGTCCCATGGCTCCATTC |
| CDC42 | AAGCCGTCGTTCTGCTGCTTGG | TGGGTGACCGACTAAGGCTGCAA |
| DHRS9 | TGGTTACAGTCTGGATAAGACACTGC | ATGGCACCACAGCCAGAACACC |
| CD86 | TCACAGTGCCTGAAGATTGGTCA | GAGGGTTCTTGATTGCTTGCTT |
| NOS2 | CTGGAGGTCAATTCCTGGAAAA | TCCCCGTTTCCTTCCTGACAGCAG |
| CD163 | CAGAGGGAGCAGATCTGAGCCTGA | TGGCTGTGACGGCAGTTGGACA |
| CD206 | TCATCGCTCGTTGTTCCTTCCCTGT | GGACCTAAGACGCGGGGAGACATCA |
| GAPDH | GCAATGCCTCCTGCACCACCAA | TGGGAAAGCCAGTCCCCAGA |

Table S1. Primer sequence
