# Supplementary material for: Serum proteomics identify CSF1R as a novel biomarker for postoperative recurrence in chronic rhinosinusitis with nasal polyps
Source: World Allergy Organ J. 2024 Mar 2;17(3):100878. doi: 10.1016/j.waojou.2024.100878 (PMC10914524; doi:10.1016/j.waojou.2024.100878)
Supplement: Multimedia component 2 [file mmc2.docx]

|  | non-Recurrence | Recurrence | P |
| --- | --- | --- | --- |
| Number, (n) | 10 | 6 |  |
| Male, n (%) | 6 (60.0) | 3 (30.0) | 1.000 |
| Age, years | 38.0 (34.3, 45.8) | 43.0 (27.0, 51.0) | 0.317 |
| BMI, kg/m^2^ | 22.2 (19.7, 26.1) | 21.5 (20.5, 23.0) | 0.518 |
| Allergic rhinitis, n (%) | 2 (20.0) | 2 (33.3) | 0.604 |
| Asthma, n (%) | 1 (10.0) | 2 (33.3) | 0.518 |
| Lund-MacKay score | 12.5 (9.8, 14.0) | 12.0 (11.0, 14.0) | 0.858 |
| Lund-Kennedy score | 6.5 (5.8, 7.0) | 6.0 (5.0, 7.0) | 0.937 |
| Follow-up time, months | 22.5 (15.0, 24.0) | 12.0 (11.0, 18.0) | <0.001 |

Table S2. Characteristics of CRSwNP patients in the discovery cohort

CRSwNP, chronic rhinosinusitis with nasal polyps; BMI, body mass index.
