# Supplementary material for: Serum proteomics identify CSF1R as a novel biomarker for postoperative recurrence in chronic rhinosinusitis with nasal polyps
Source: World Allergy Organ J. 2024 Mar 2;17(3):100878. doi: 10.1016/j.waojou.2024.100878 (PMC10914524; doi:10.1016/j.waojou.2024.100878)
Supplement: Multimedia component 3 [file mmc3.docx]

Table S3. Top 3 differently expressed proteins between the two groups

| Protein | Gene name | Regulation | FC | Log_2_FC | P |
| --- | --- | --- | --- | --- | --- |
| Colony-stimulating factor 1 receptor | CSF1R | up | 3.13 | 1.65 | <0.001 |
| Cell division control protein 42 | CDC42 | up | 2.95 | 1.56 | 0.001 |
| C-C motif chemokine 5 | CCL5 | up | 2.74 | 1.45 | <0.001 |
| Ubiquitin-conjugating enzyme E2 variant 1 | UBE2V1 | down | 0.46 | -1.09 | 0.004 |
| Pregnancy zone protein | PZP | down | 0.34 | -1.58 | 0.034 |
| Dehydrogenase/reductase 9 | DHRS9 | down | 0.33 | -1.60 | 0.035 |

FC, fold change
